# Supplementary material for: Development of FluoAHRL: A Novel Synthetic Fluorescent Compound That Activates AHR and Potentiates Anti-Inflammatory T Regulatory Cells
Source: Molecules. 2024 Jun 23;29(13):2988. doi: 10.3390/molecules29132988 (PMC11243367; doi:10.3390/molecules29132988)
Supplement: Supplementary file 1 [file molecules-29-02988-s001.zip › molecules-3027937-supplementary.pdf]

Supplementary data

Development of FluoAHRL: A Novel Synthetic Fluorescent Compound That Activates AHR and Potentiates Anti-Inflammatory T Regulatory Cells

Natalija Jonić [1], Ivan Koprivica [1], Christos M. Chatzigiannis [2], Antonis D. Tsiailanis [2], Stavroula G. Kyrkou [2], Eleftherios Paraskevas Tzakos [3], Aleksandar Pavić [4], Mirjana Dimitrijević [1], Andjelina Jovanović [5], Milan B. Jovanović [5,6], Sérgio Marinho [7,8], Inês Castro-Almeida [7,8], Vesna Otašević 9, Pedro Moura-Alves [7,8,*], Andreas G. Tzakos [2,10,*] and Ivana Stojanović [1,*]

1 Department of Immunology, Institute for Biological Research "Siniša Stanković"—National Institute of the Republic of Serbia, University of Belgrade, 11108 Belgrade, Serbia; natalija.jonic@ibiss.bg.ac.rs (N.J.); ivan.koprivica@yahoo.com (I.K.); mirjana.dimitrijevic@ibiss.bg.ac.rs (M.D.)

2 Section of Organic Chemistry & Biochemistry, Department of Chemistry, University of Ioannina,
45110 Ioannina, Greece; cmchatzigiannis@gmail.com (C.M.C.);
antonis.tsiailanis@gmail.com (A.D.T.);
stavroylakyrkoy@gmail.com (S.G.K.)

3 Department of Biology, National and Kapodistrian University of Athens, 15772 Athens, Greece;
etzakos@gmail.com

4 Laboratory for Microbial Molecular Genetics and Ecology, Institute for Molecular Genetics and Genetic Engineering, University of Belgrade, 11000 Belgrade, Serbia; pavicaleksandarr@gmail.com

5 Department of Otorhinolaryngology with Maxillofacial Surgery, Clinical Hospital Center "Zemun",
11080 Belgrade, Serbia; andjelinakjosevski@yahoo.com (A.J.); majov@eunet.rs (M.B.J.)

6 Faculty of Medicine, University of Belgrade, 11000 Belgrade, Serbia

7 Instituto de Biologia Molecular e Celular, (IBMC), Universidade do Porto, 4200-135 Porto, Portugal;
smarinho@i3s.up.pt (S.M.); ialmeida@i3s.up.pt (I.C.-A.)

8 Instituto de Investigação e Inovação em Saúde (i3S), Universidade do Porto, 4200-135 Porto, Portugal

9 Department of Molecular Biology, Institute for Biological Research "Siniša Stanković"—National Institute of the Republic of Serbia, University of Belgrade, 11108 Belgrade, Serbia; vesna@ibiss.bg.ac.rs

10 Institute of Materials Science and Computing, University Research Center of Ioannina (URCI),
45110 Ioannina, Greece

* Correspondence: pmouraalves@i3s.up.pt (P.M.-A.); atzakos@uoi.gr (A.G.T.); ivana@ibiss.bg.ac.rs (I.S.)

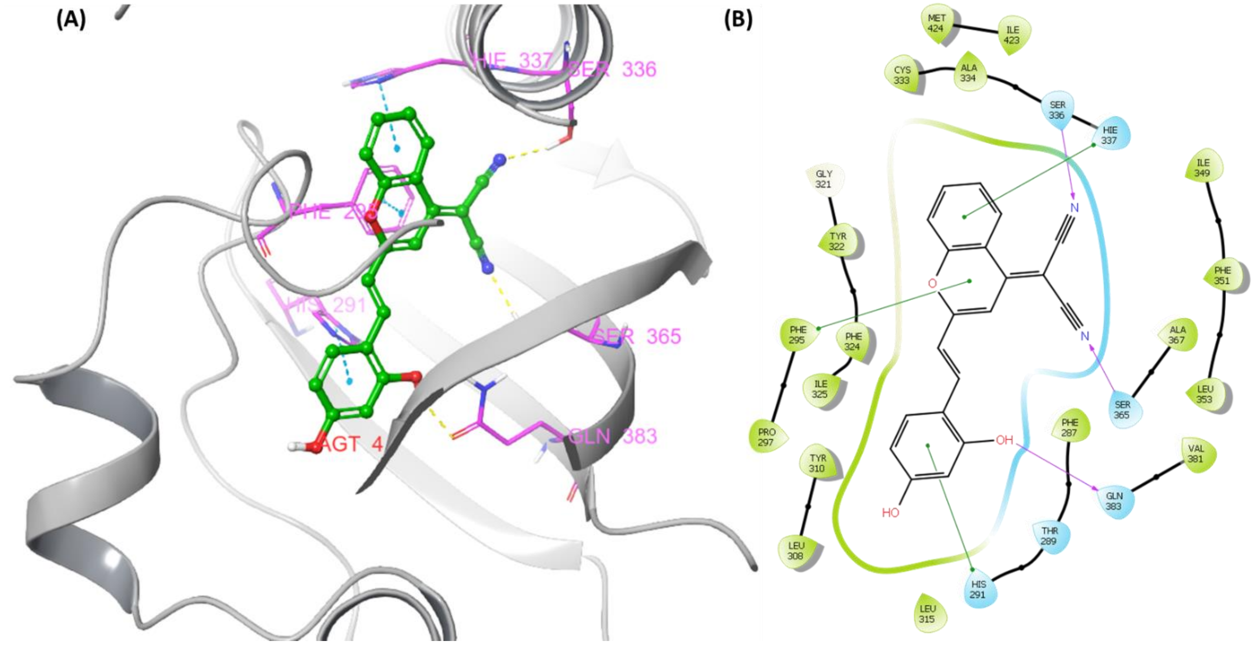

Figure S1. (A) Docking pose for AGT-4 bound to AHR PAS-B. (B) AGT-4–PAS-B ligand-protein interaction diagram.

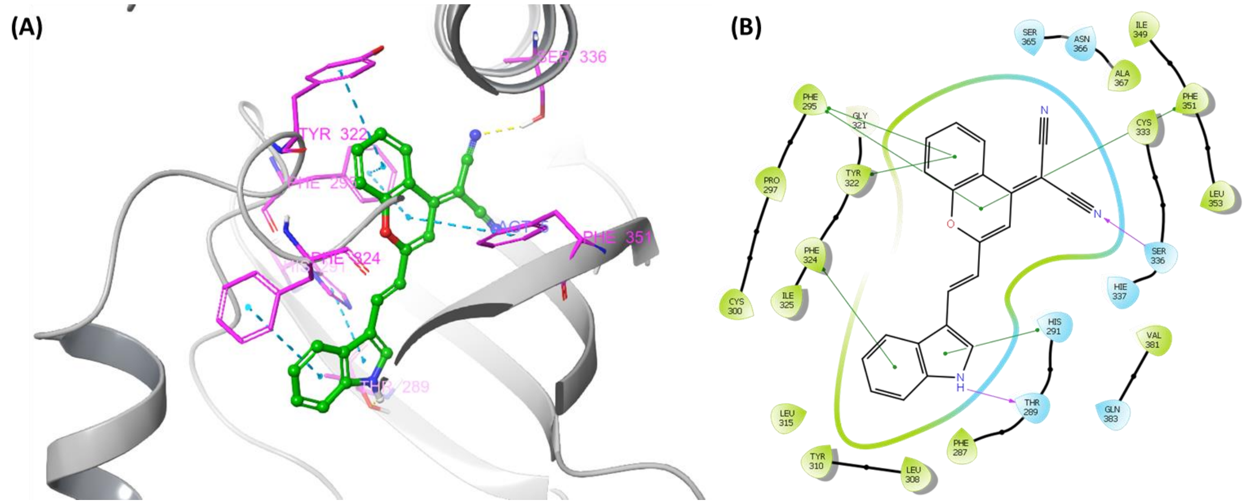

Figure S2. (A) Docking pose for AGT-5 bound to AHR PAS-B. (B) AGT-5–PAS-B ligand-protein interaction diagram.

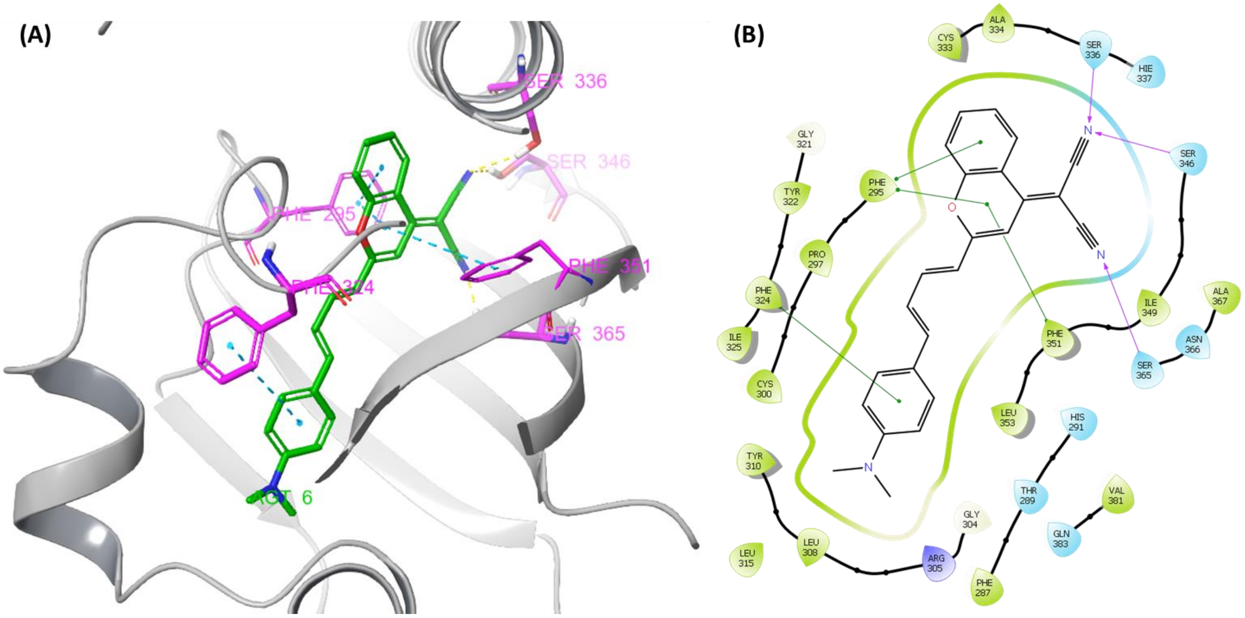

Figure S3. (A) Docking pose for AGT-6 bound to AHR PAS-B. (B) AGT-6–PAS-B ligand protein interaction diagram.

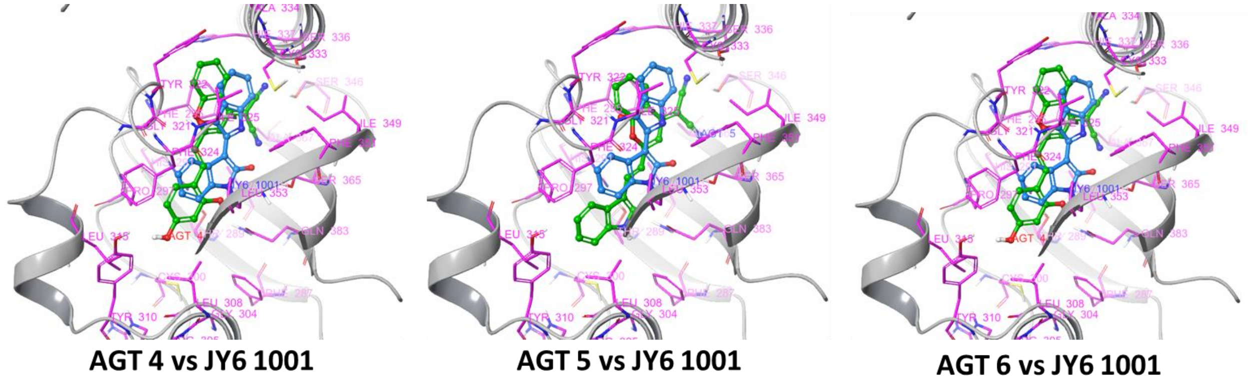

Figure S4. Superposition of the docked poses of AGT-4, AGT-5, and AGT-6 (colored in green) bound to AHR PAS-B with the X-ray structure of indirubin (colored in cyan, (pdbid: 7ZUB).

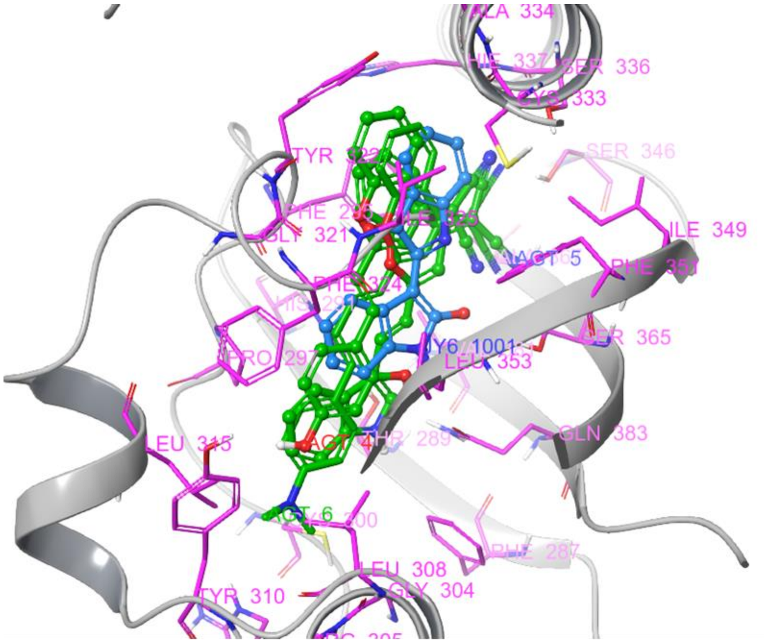

AGT 6 vs AGT 4 vs AGT 5 vs JY6 1001

Figure S5. The docked poses of all AGT analogs overlaid with the X-ray structure of indirubin, attached to AHR PAS-B (pdbid: 7ZUB).

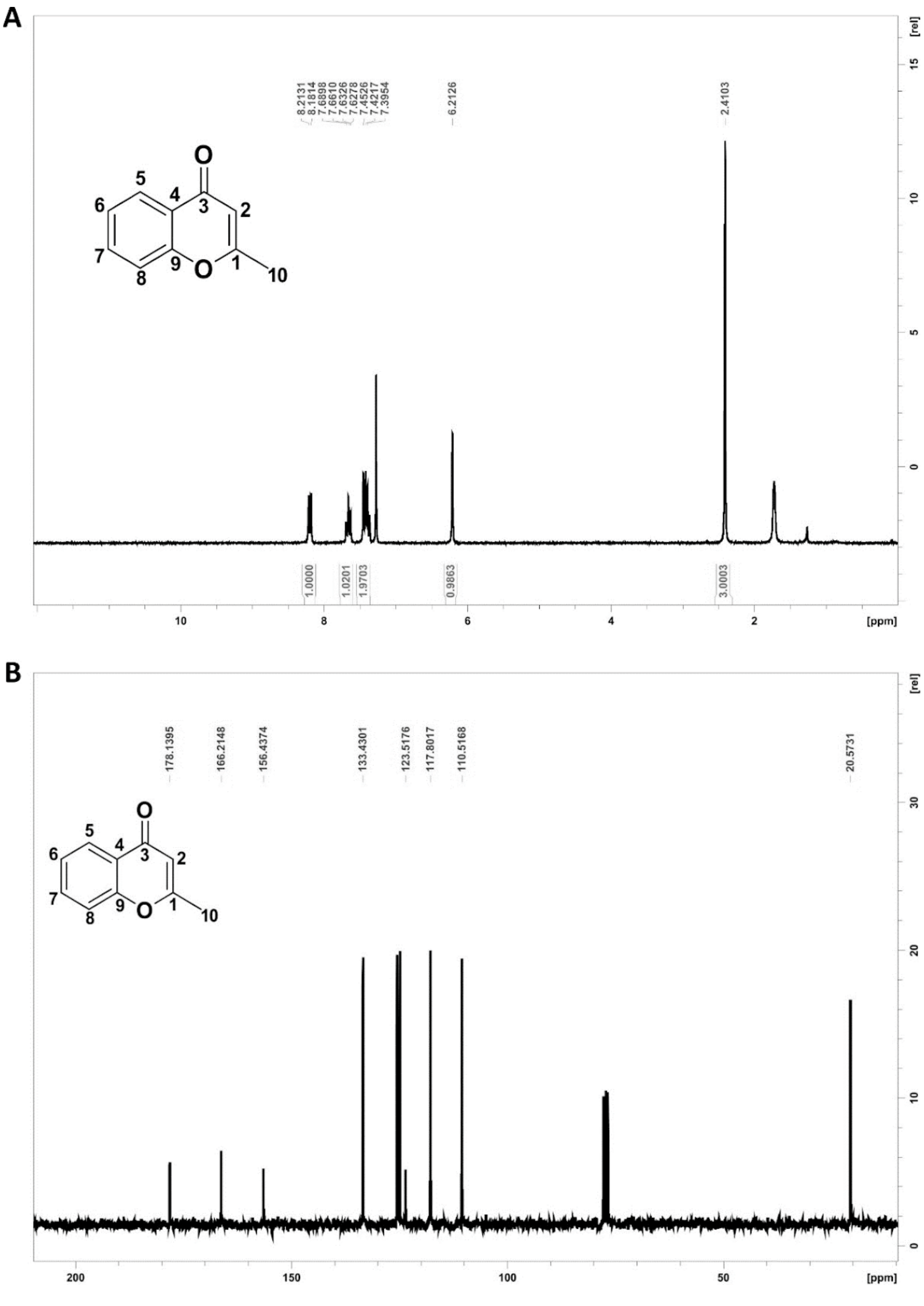

Figure S6. (A) ^1H NMR spectrum of (1) in $CDCl_3$. (B) ^{13}C NMR spectrum of (1) in $CDCl_3$.

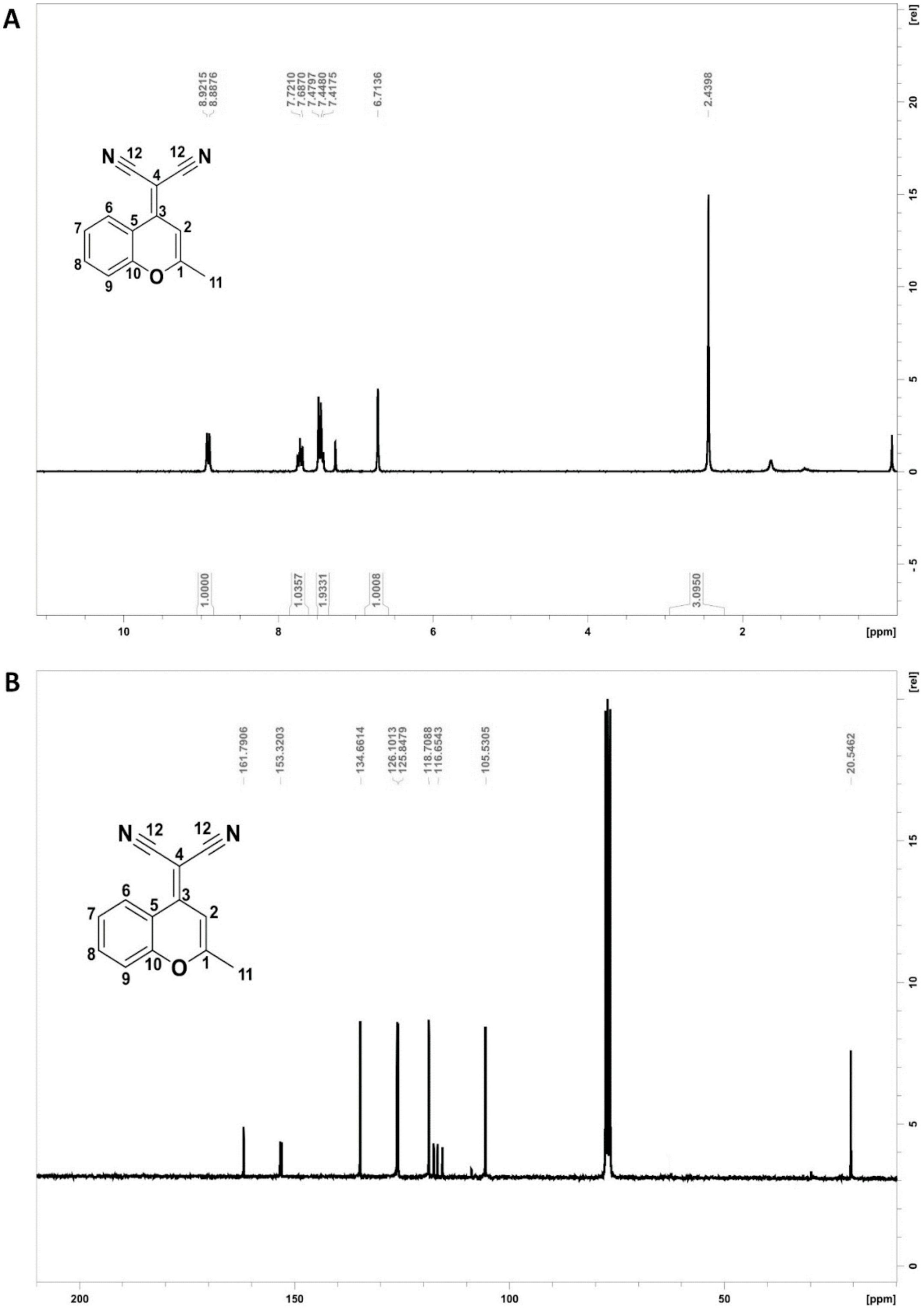

Figure S7. (A) ^{1}H NMR spectrum of (2) in $CDCl_3$. (B) ^{13}C NMR spectrum of (2) in $CDCl_3$.

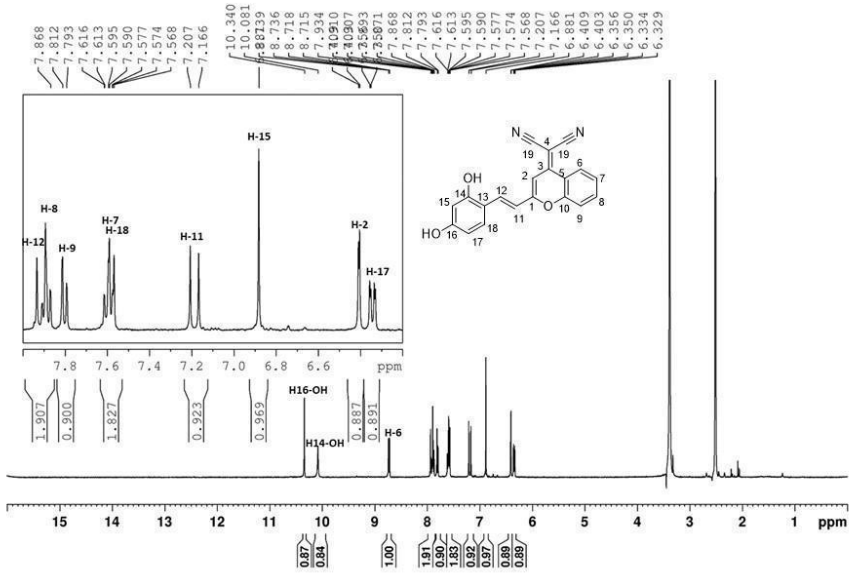

Figure S8. ^{1}H NMR spectrum of AGT-4 in DMSO-d$_6$.

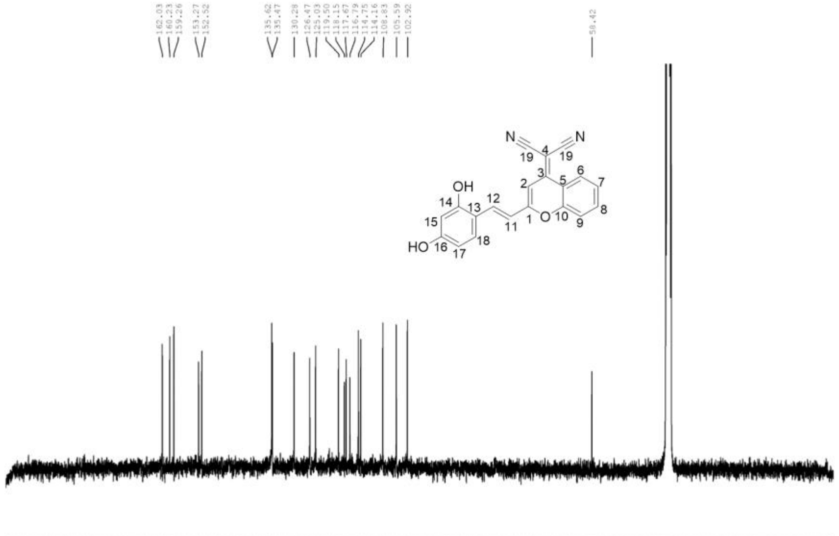

Figure S9. ^{13}C NMR spectrum of AGT-4 in DMSO-d$_6$.

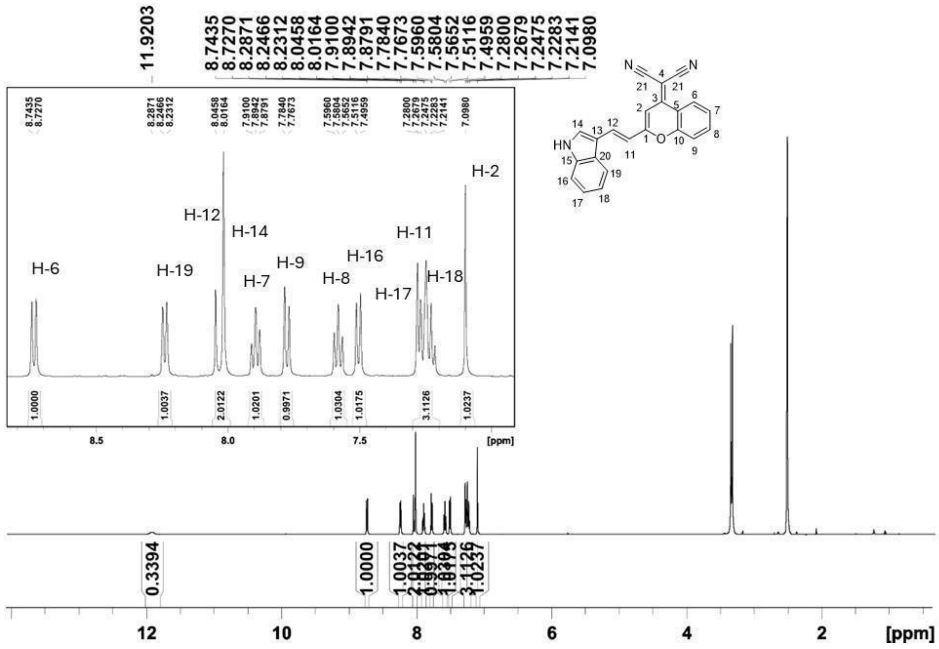

Figure S10. ^{1}H NMR spectrum of AGT-5 in DMSO-d_6.

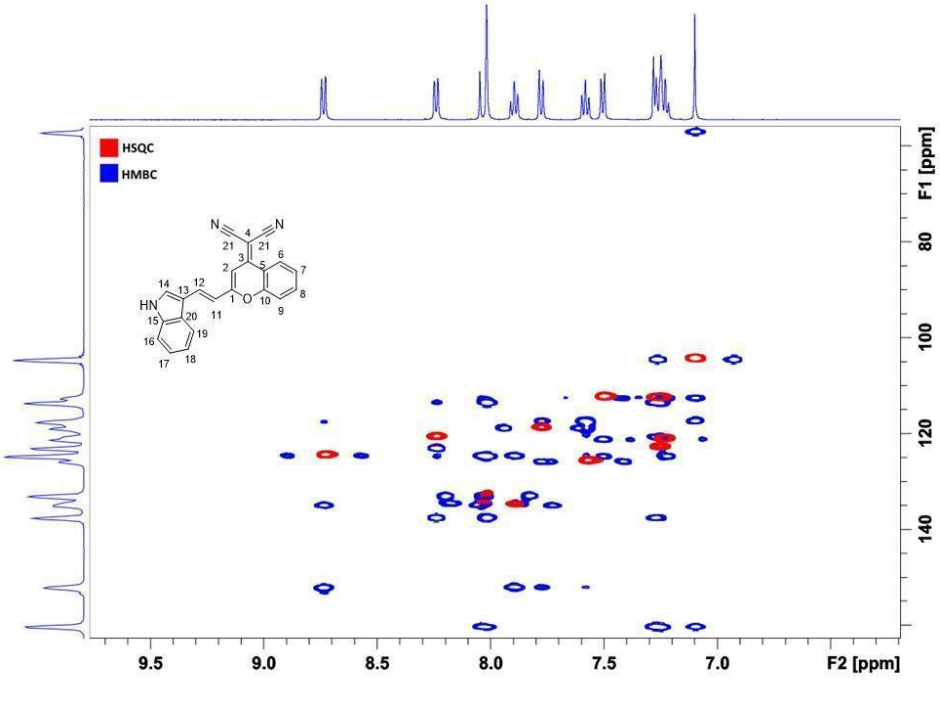

Figure S11. Overlay of 2D NMR spectra, HSQC (red)-HMBC (blue), of AGT-5 in DMSO-d_6.

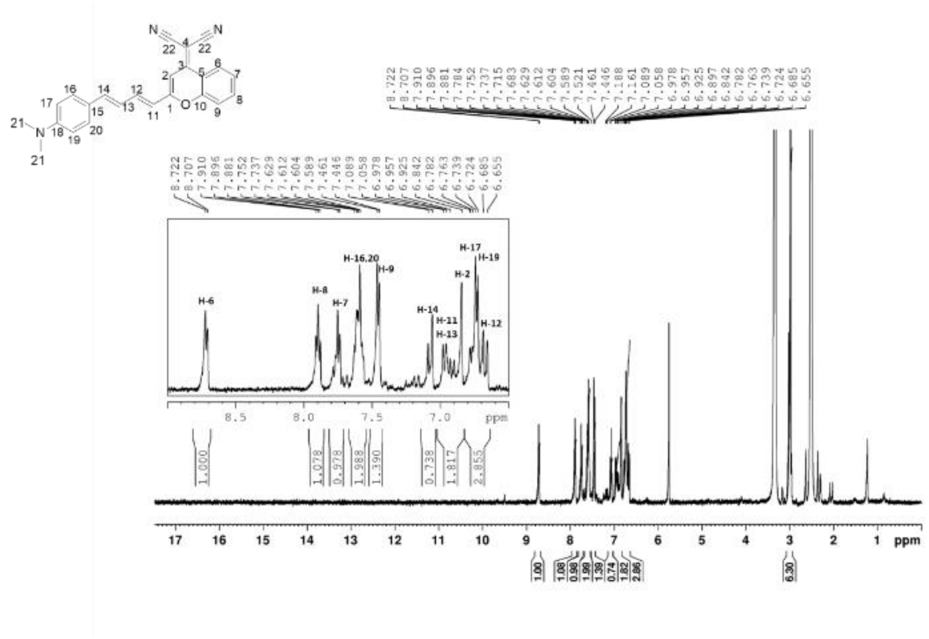

Figure S12. ^{1}H NMR spectrum of AGT-6 in DMSO-d_6.

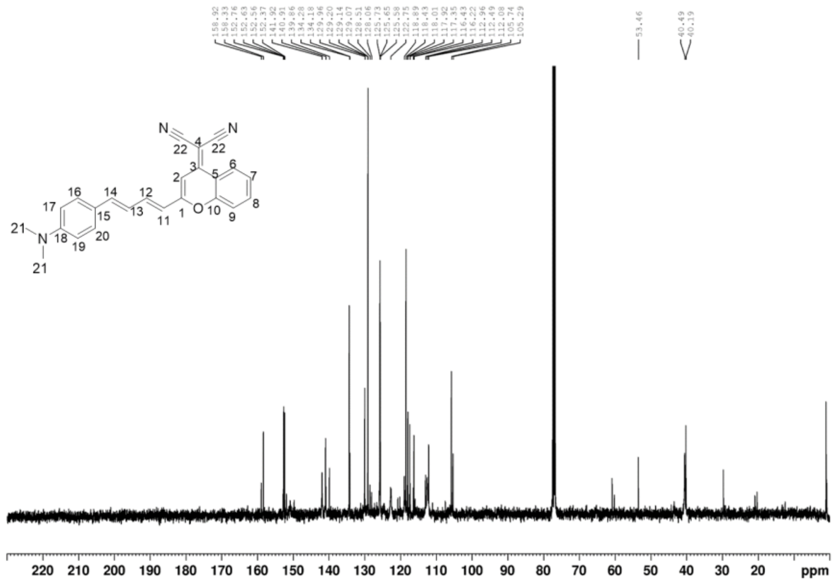

Figure S13. ^{13}C NMR spectrum of AGT-6 in DMSO-d_6.

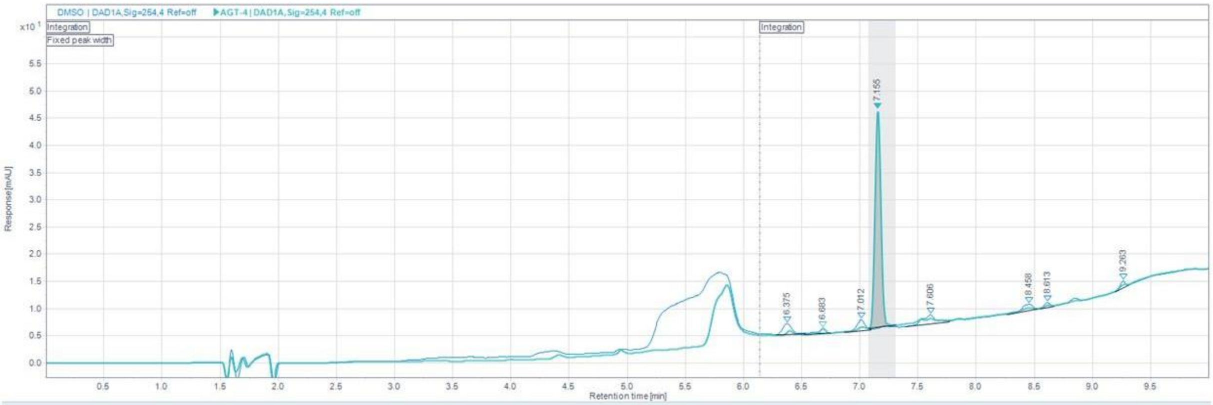

Injection Results

Peaks | Summary

#	Name	Signal description	RT (min)	Area (mAU·s)	Area%	Height (mAU)	Height%	Amount	Concentration	Start time (min)	End time (min)
1		DAD1A,Sig=254,4 Ref=off	6.683	3.990	2.862	0.908	2.22			6.531	6.752
2		DAD1A,Sig=254,4 Ref=off	7.155	135.435	97.138	39.992	97.78			7.078	7.314

Figure S14. Analytical HPLC of the compound AGT-4 where it appears to elute at retention time: 7.155 with a percent determination of 97%.

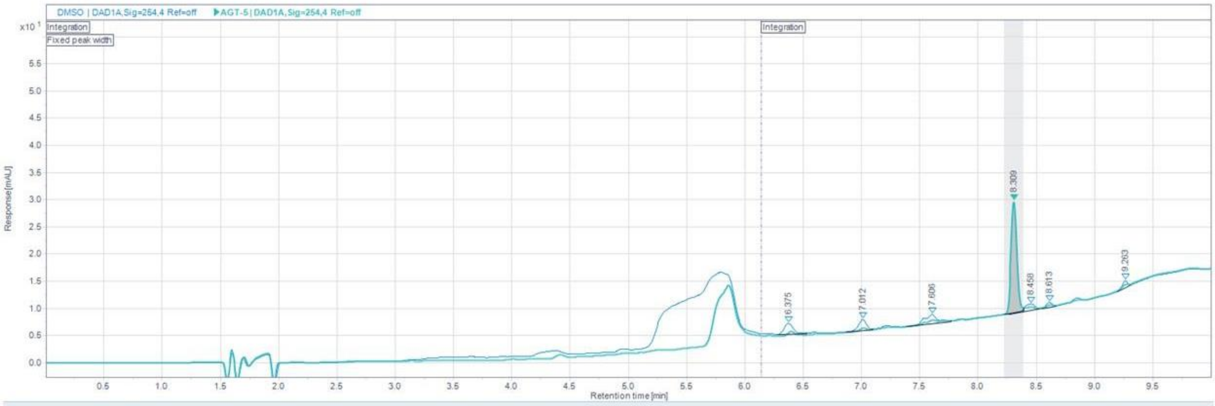

Injection Results

Peaks | Summary

#	Name	Signal description	RT (min)	Area (mAU·s)	Area%	Height (mAU)	Height%	Amount	Concentration	Start time (min)	End time (min)
1		DAD1A,Sig=254,4 Ref=off	8.309	72.183	100.000	20.527	100.00			8.225	8.394

Figure S15. Analytical HPLC of the compound AGT-5 where it appears to elute at retention time: 8.309 with a percent determination of 100%.

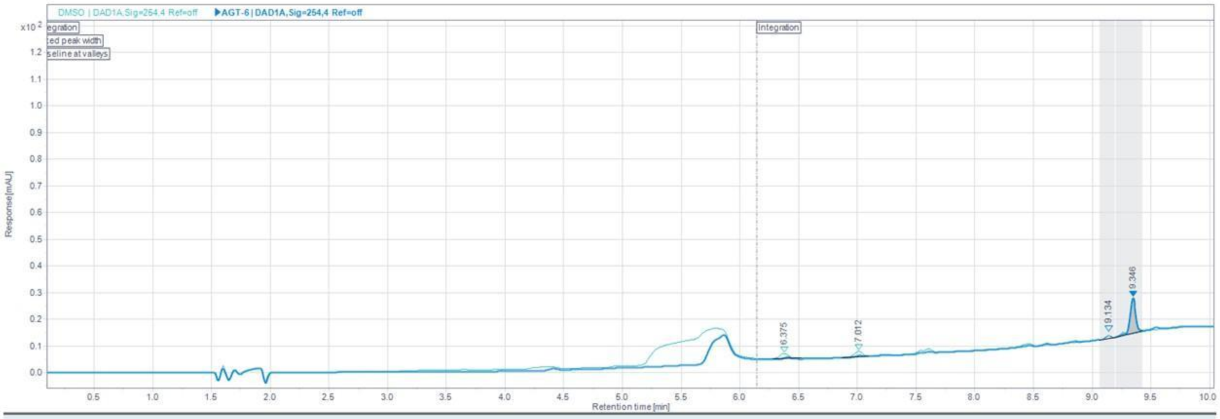

Injection Results

Peaks Summary

#	Name	Signal description	RT (min)	Area (mAU·s)	Area%	Height (mAU)	Height%	Amount	Concentration	Start time (min)	End time (min)
1		DAD1A,Sig=254,4 Ref=off	9.134	5.071	4.659	1.986	11.13			9.103	9.193
2		DAD1A,Sig=254,4 Ref=off	9.346	103.774	95.341	15.862	88.87			9.205	9.537

Figure S16. Analytical HPLC of the compound AGT-6 where it appears to elute at retention time: 9.346 with a percent determination of 95%.

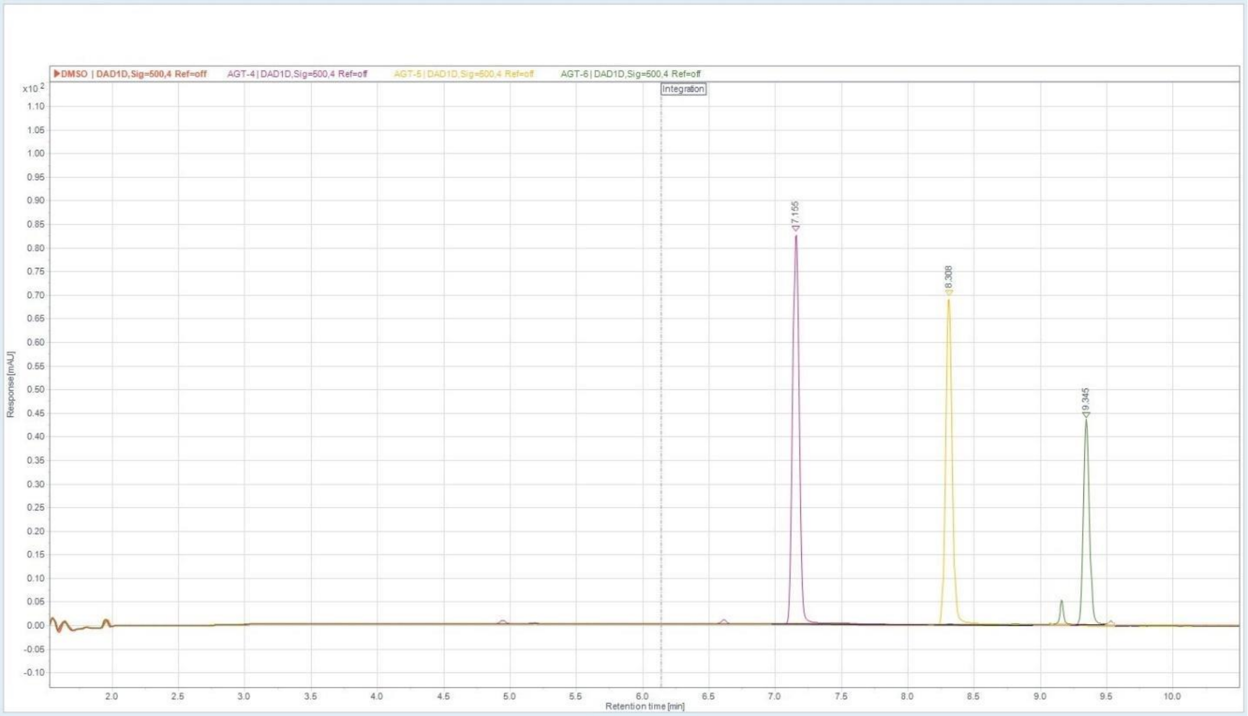

Figure S17. Chromatogram of the overlaid traces for the blank, AGT-4, AGT-5, and AGT-6. Detector is set at 500 nm.

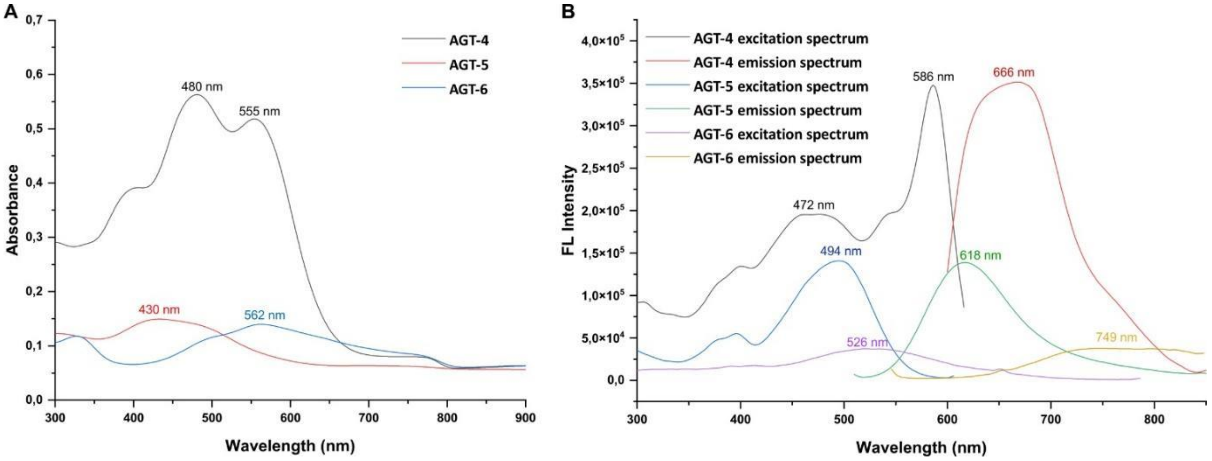

Figure S18. Spectral properties of compounds AGT-4, AGT-5, AGT-6 (10 μM), (A) Absorption and (B) fluorescence spectra, in DMSO:PBS (3:7 v/v, 10 mM, pH 7.4) at 37°C [step 2, ExBw: 5, EmBw: 5].

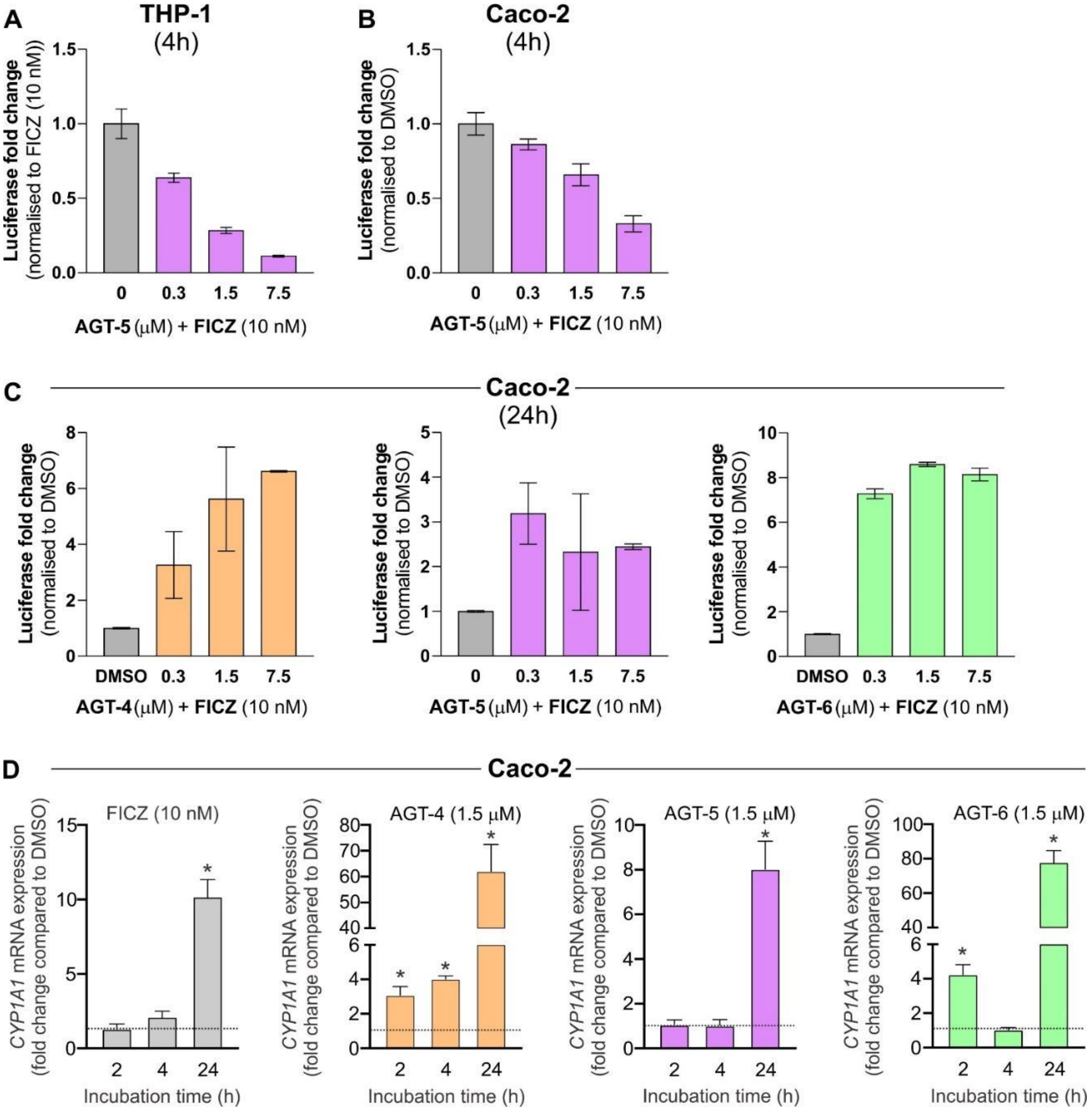

Figure S19. The effect of putative FluoAHRL on AHR transcriptional activity in cells cultured in tryptophan-free medium. THP-1 (A) or Caco-2 (B, C) cells were cultured in the cell medium without tryptophan in the presence of FICZ (10 nM) and growing concentrations of AGT-5, AGT-4 and AGT-6 for the indicated time periods. (D) CYP1A1 mRNA expression in Caco-2 cells cultured in tryptophan-free medium was measured by qRT-PCR after 2 h, 4 h or 24 h of treatment, normalized to the expression of GAPDH and then normalized to the values obtained in DMSO-treated cells at indicated time points.

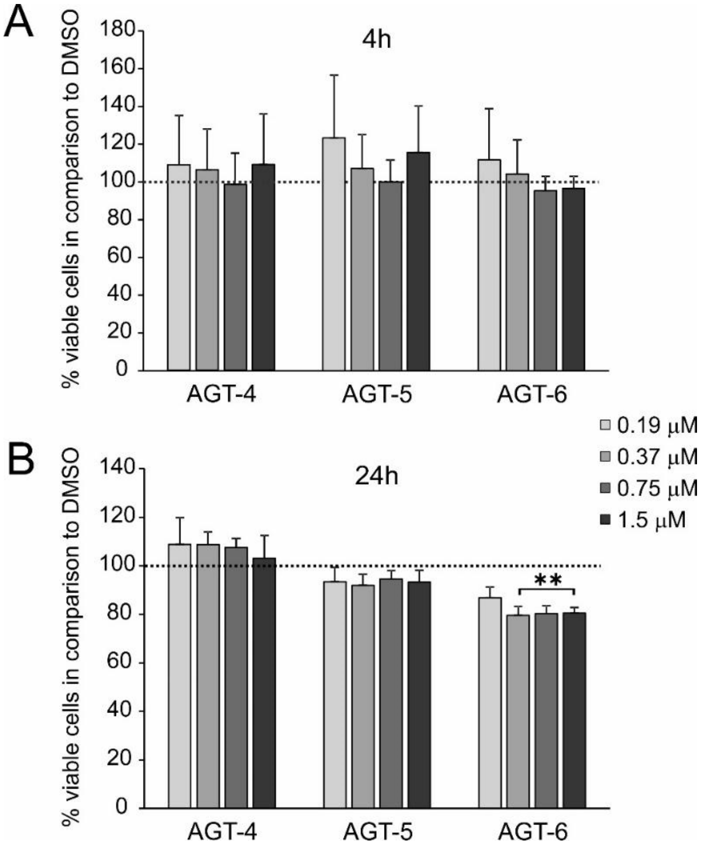

Figure S20. The effect of FluoAHRL on macrophage viability *in vitro*. Peritoneal macrophages were cultivated for 4 h (A) or 24 h (B) in the presence of growing concentrations of AGT-4, AGT-5 and AGT-6, after which the viability was measured by the MTT assay. **p<0.01 was considered as a statistically significant difference between AGT-6-treated cells and DMSO-treated cells.

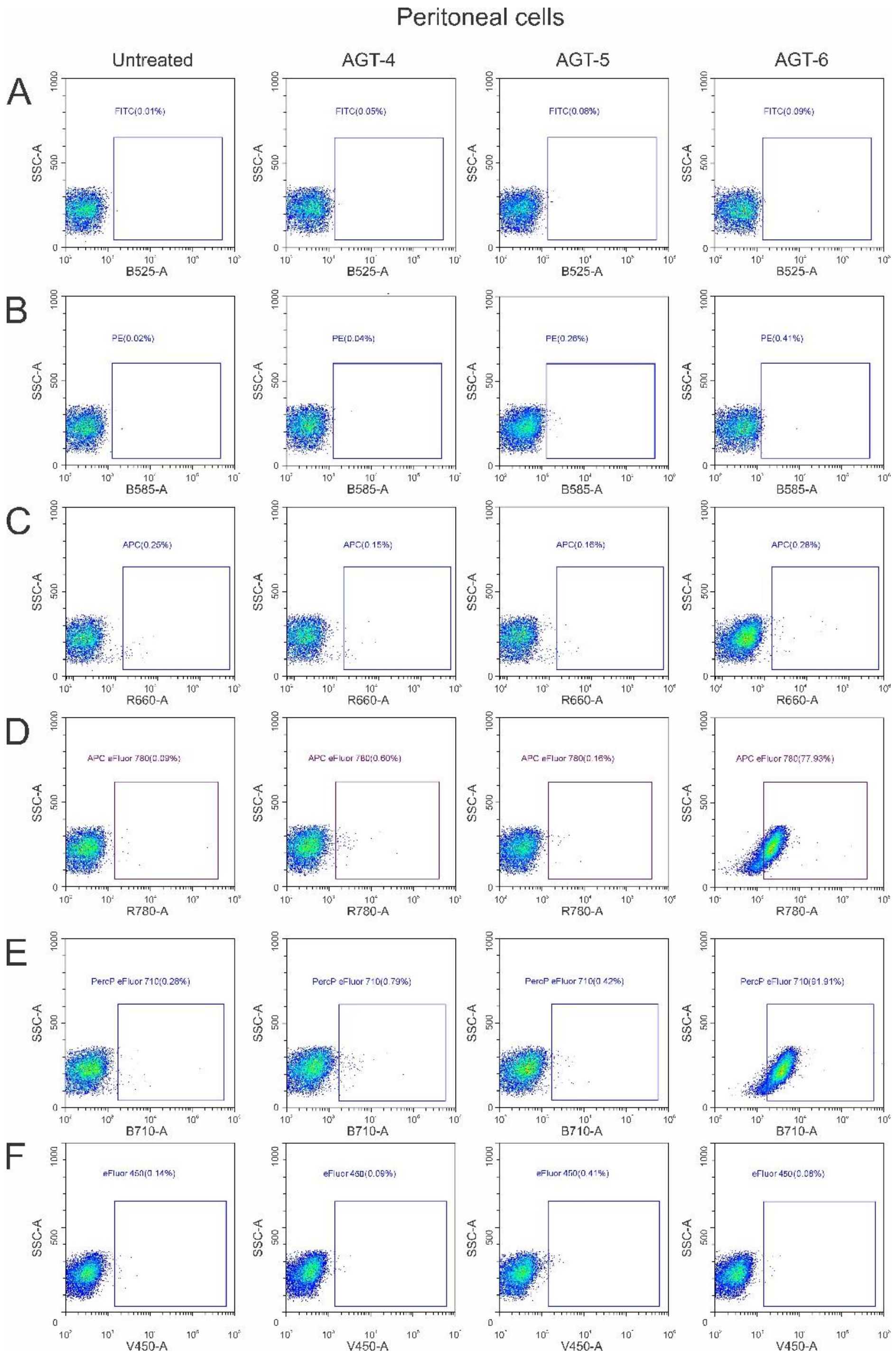

Figure S21. Peritoneal cells under FluoAHRL exposure. Exposure of peritoneal cells to the FluoAHRL (1.5 μM) for 48 h and detection of fluorescent signal in all channels that were used for detection of specific staining with antibodies. (A) FITC channel (B525 nm). (B) PE channel (B585 nm). (C) APC channel (R660 nm). (D) APC-eF780 channel (R780 nm). (E) PercP-eF710 channel (B710 nm). (F) eFluor450 channel (V450 nm).

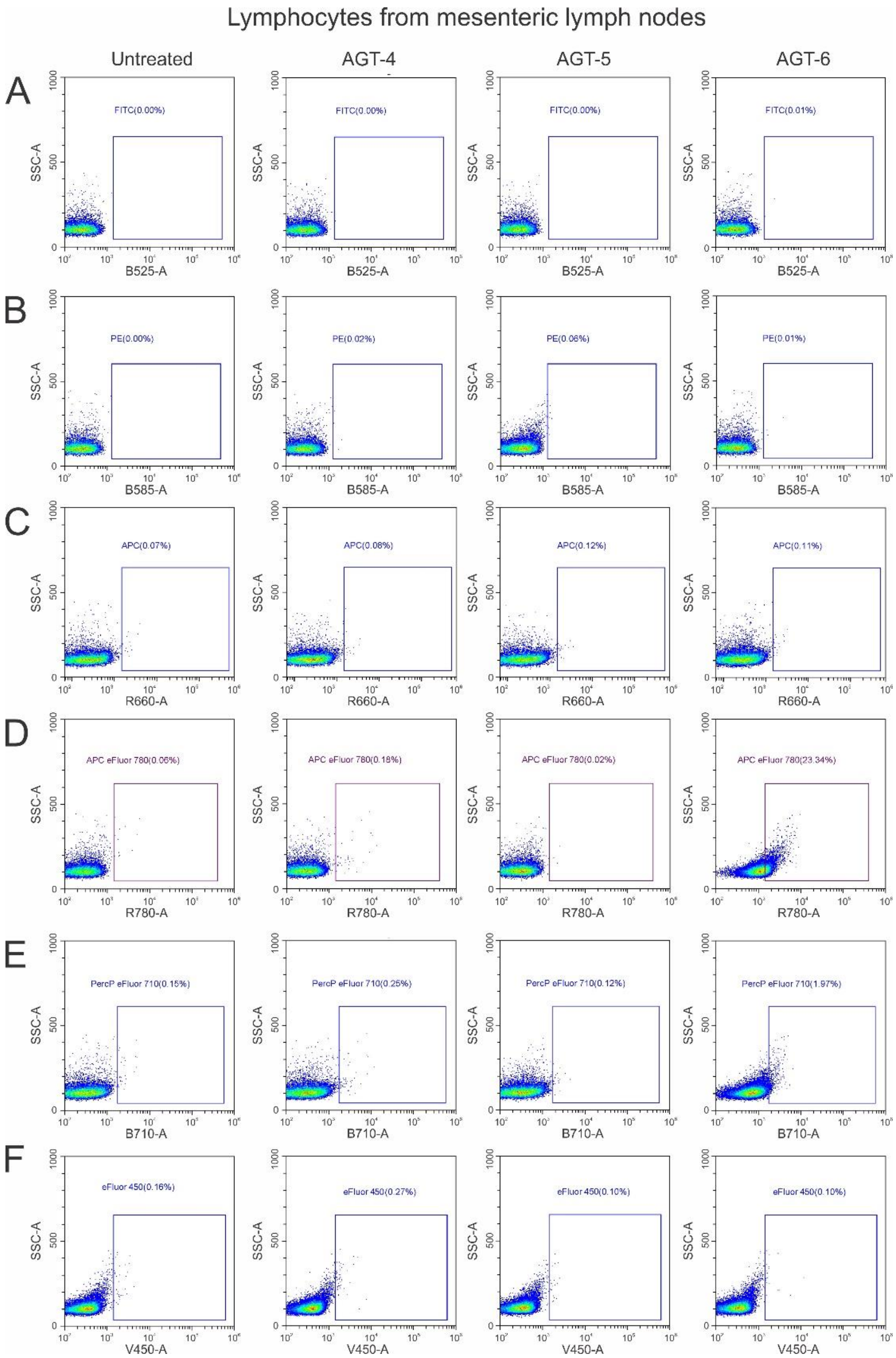

Figure S22. Mesenteric lymph node cells under FluoAHRL exposure. Exposure of mesenteric lymph node cells to the FluoAHRL (1.5 µM) for 48 h and detection of fluorescent signal in all channels that were used for detection of specific staining with antibodies. (A) FITC channel (B525 nm). (B) PE channel (B585 nm). (C) APC channel (R660 nm). (D) APC-eF780 channel (R780 nm). (E) PercP-eF710 channel (B710 nm). (F) eFluor450 channel (V450 nm).

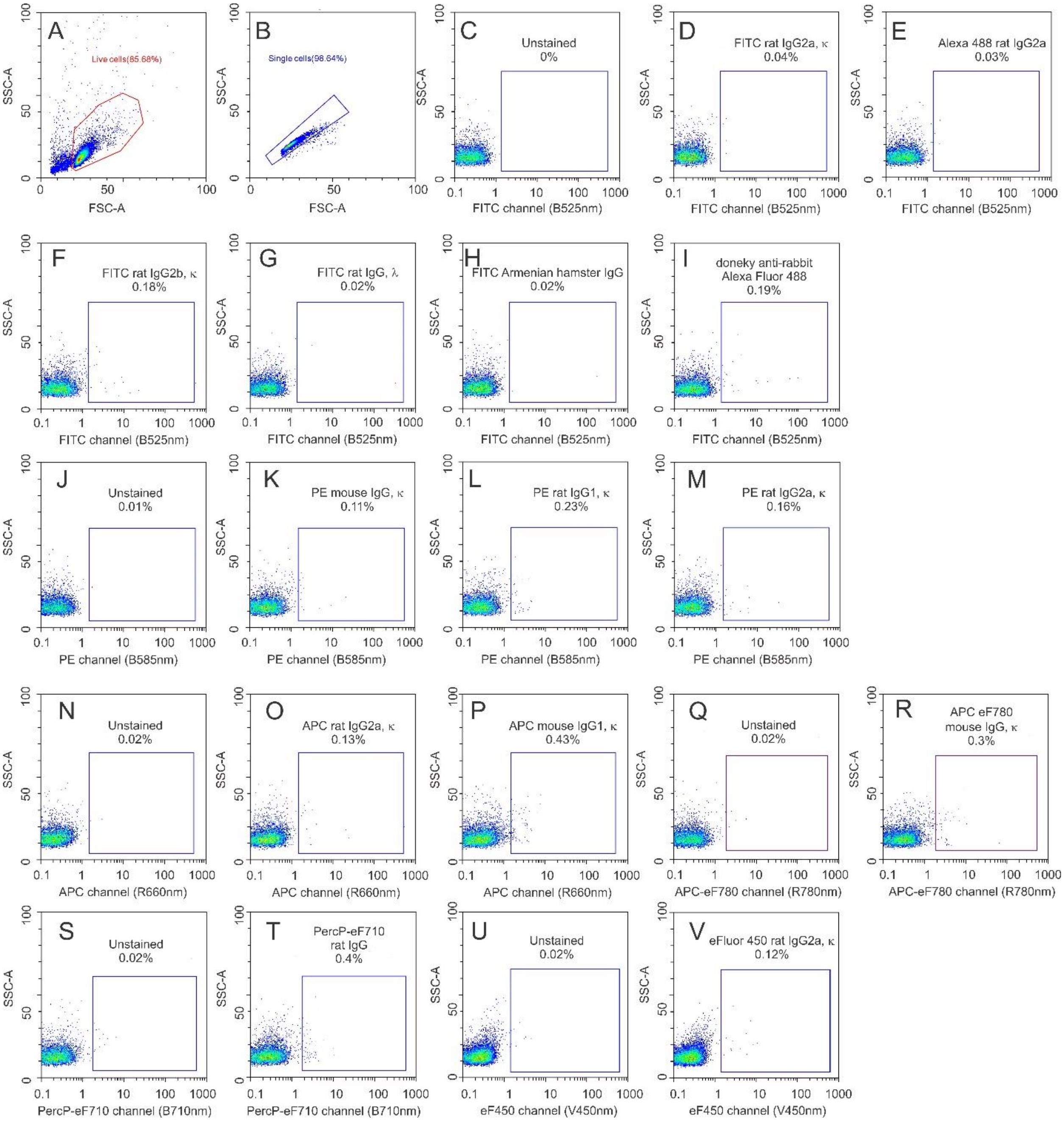

Figure S23. Staining of lymphocytes from mesenteric lymph nodes with adequate isotype controls. (A) Representation of cells in FSC-A and SSC-A plot. (B) Representation of single cells. (C-I) Representation of isotype controls for FITC-conjugated antibodies. (J-M) Representation of isotype controls for PE-conjugated antibodies. (N-P) Representation of isotype controls for APC or eFluor660-conjugated antibodies. (Q-R) Representation of isotype controls for APC eF780-conjugated antibodies. (S-T) Representation of isotype controls for PercP-eF710-conjugated antibodies. (U-V) Representation of isotype controls for eF450-conjugated antibodies.

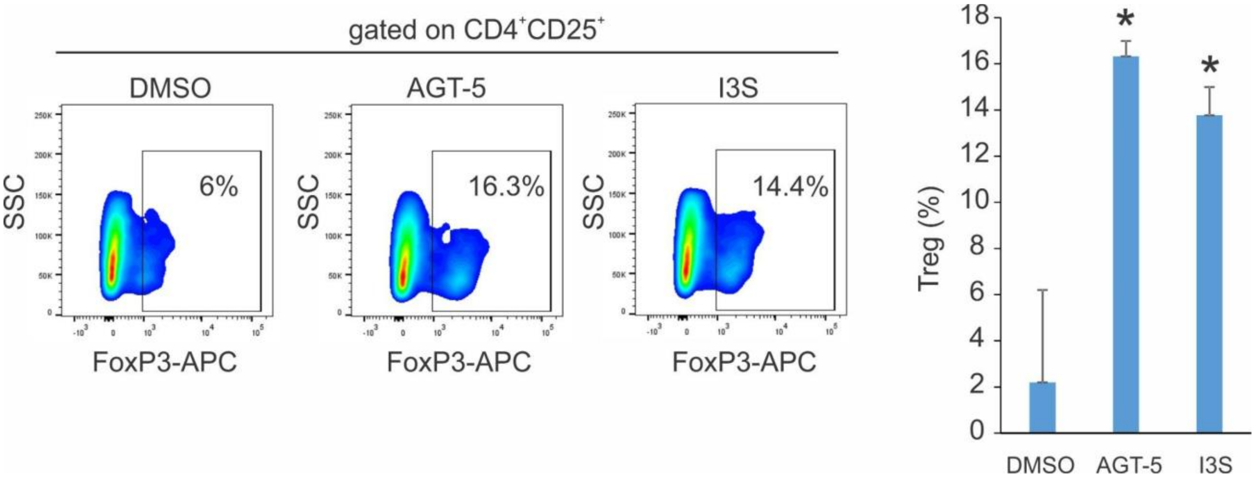

Figure S24. The comparison between I3S and AGT-5 effects on Treg. $CD4^+$ cells were exposed to anti-CD3 and anti-CD28 antibodies and treated with I3S (1.5 μM), AGT-5 (1.5 μM) or DMSO for 48 h. Treg ($CD4^+CD25^+FoxP3^+$) were detected by flow cytometry. Representative plots are shown. *$p<0.05$ was considered as a statistically significant difference between I3S-treated or AGT-5-treated cells vs. DMSO-treated cells.

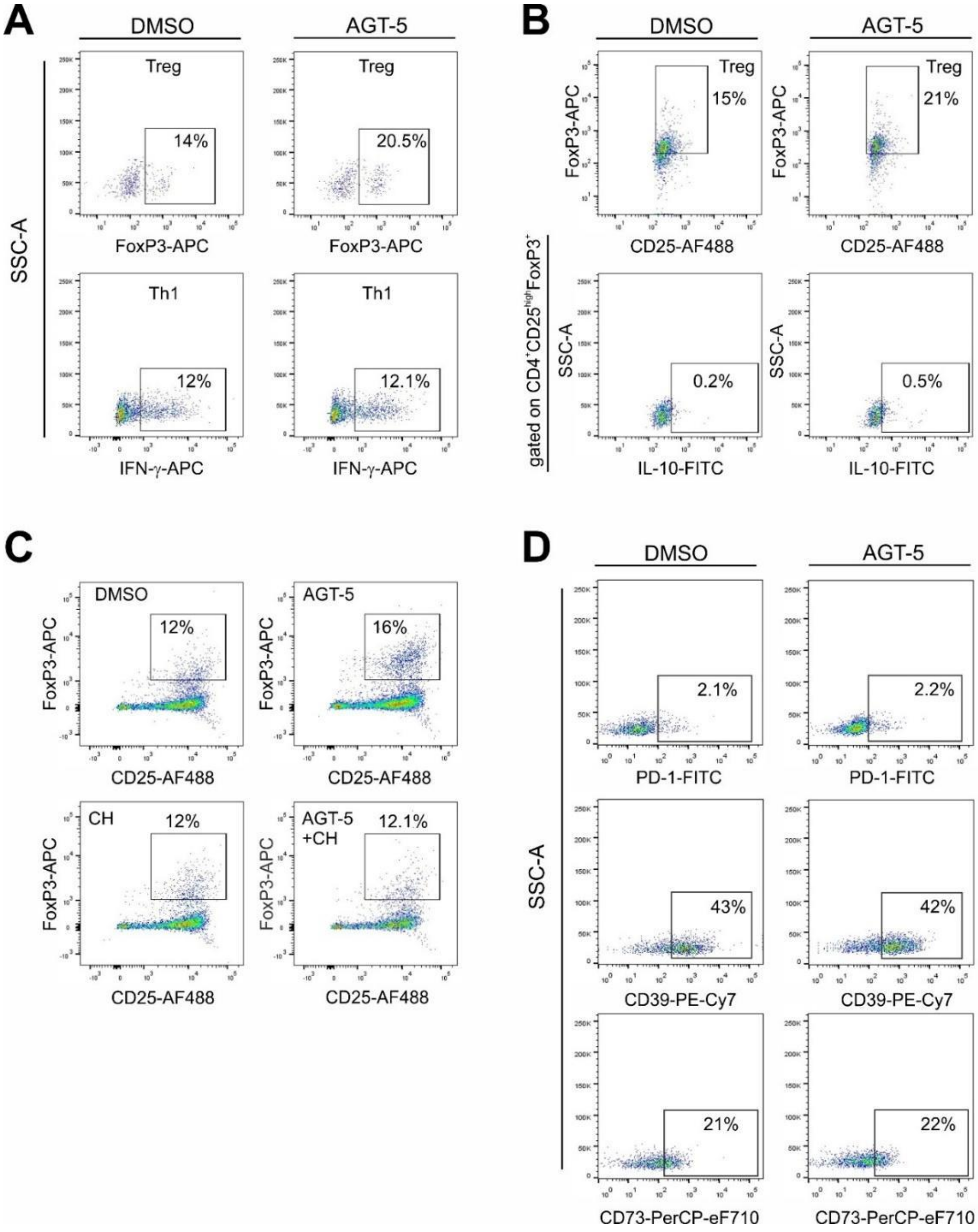

Figure S25. Representative flow cytometry plots for T cells *in vitro*. (A) $CD4^+$ were exposed to anti-CD3 and anti-CD28 antibodies and treated with AGT-5 (1.5 μM) or DMSO for 48 h, and Th1 (IFN-γ^+) and Treg ($CD25^{high}FoxP3^+$) profiles were evaluated. (B) Sorted $CD4^+CD25^{high}$ were treated with AGT-5 for 48 h in the presence of the "complete" stimulation cocktail and the proportions of Treg ($CD4^+CD25^{high}FoxP3^+$) and IL-10^+ Treg were determined by flow cytometry. (C) $CD4^+CD25^-$ cells were stimulated by anti-CD3 and anti-CD28 antibodies and treated with AGT-5 (1.5 μM) in the presence or absence of AHR inhibitor CH-223191 (CH, 1.5 μM) and the proportion of Treg was determined ($CD4^+CD25^{high}FoxP3^+$). (D) Sorted $CD4^+CD25^{high}$ were treated with AGT-5 for 48 h in the presence of the "complete" stimulation cocktail and the proportions of Treg expressing PD-1, CD39, and CD73 were ascertained.

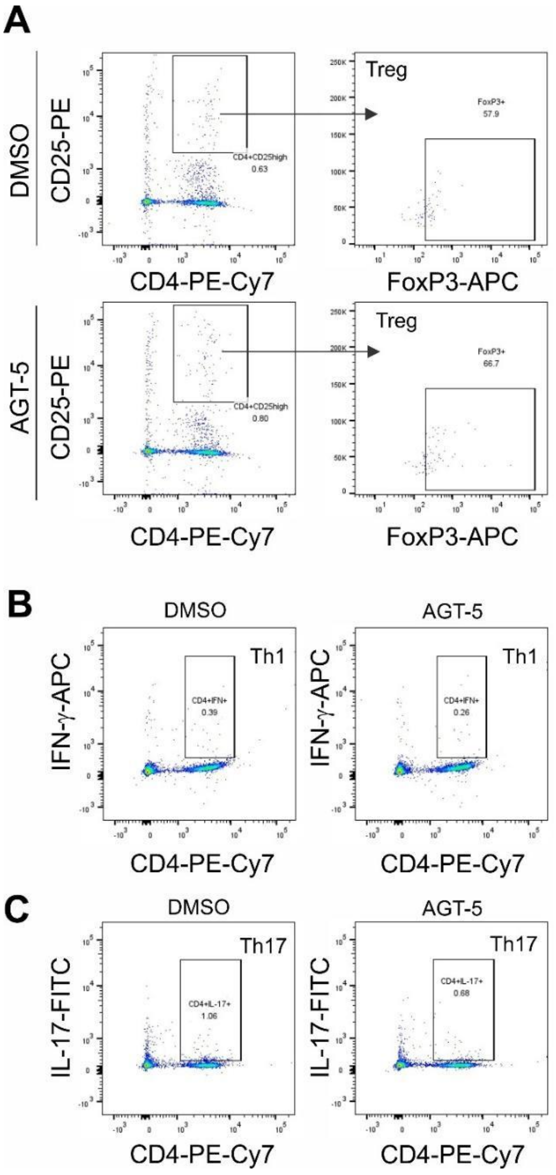

Figure S26. Representative flow cytometry plots and gating strategy for Treg (A), Th1 (B), and Th17 cells (C) (*ex vivo* analysis).

Table S1. Calculated pharmacokinetic parameters for AGT-4, AGT-5, and AGT-6.

**Pharmacokinetics**			
	**AGT-4**	**AGT-5**	**AGT-6**
**GI absorption**	High	High	High
**BBB permeant**	No	No	Yes
**P-gp substrate**	No	No	No
**Log Kp (skin permeation)**	-5.97 cm/s	-5.41 cm/s	-5.14 cm/s

Table S2. Calculated drug-likeness parameters for AGT-4, AGT-5, and AGT-6.

**Drug-likeness**			
	**AGT-4**	**AGT-5**	**AGT-6**
**Lipinski**	Yes; 0 violation	Yes; 0 violation	Yes; 0 violation
**Ghose**	Yes	Yes	Yes
**Veber**	Yes	Yes	Yes
**Egan**	Yes	Yes	Yes
**Muegge**	Yes	Yes	Yes
**Bioavailability Score**	0.55	0.55	0.55

Table S3. Lethal and teratogenic effects observed in the I3S-treated (5 μM) zebrafish (*Danio rerio*) embryos at different hours post-fertilization (hpf).

Category	Toxicological parameters	Exposure time (hpf)				
		24	48	72	96	120
**Lethal effect**	Coagulated eggs[a]	●	●	●	●	●
	Lack of the heart beating	●	●	●	●	●
	Non-detachment of the tail	●	●	●	●	●
	Lack of somite formation	●	●	●	●	●
**Teratogenic effect**	Malformation of head	●	●	●	●	●
	Malformation of eyes[b]	●	●	●	●	●
	Malformation of sacculi/otoliths[c]	●	●	●	●	●
	Malformation of chorda	●	●	●	●	●
	Malformation of tail[d]	●	●	●	●	●
	Scoliosis	●	●	●	●	●
	Yolk edema	●	●	●	●	●
	Yolk deformation	●	●	●	●	●
	Growth retardation[e]		●	●	●	●
	Hatching			●	●	●
	Swimmbladder development					●
**Hepatotoxicity**	Yolk absorption			●	●	●
	Liver darkening			●	●	●
**Cardiotoxicity**	Pericardial edema		●	●	●	●
	Heart morphology			●	●	●
	Heart beating rate (beat/min)				●	●
**Melanocytotoxicity**	Skin pigmentation (melanization)[f]		●	●	●	●
	Melanocytes morphology[g]		●	●	●	●

[a] No clear organ structure is recognized.
[b] -Malformation of the eyes was recorded for the retardation in eye development and abnormality in shape and size.
[c] Presence of none, one or more than two otoliths per sacculus, as well as reduction and enlargement of otoliths and/or sacculi (otic vesicles).
[d] Tail malformation was recorded when the tail was bent, twisted, or shorter than control embryos as assessed by optical comparison.
[e] Growth retardation was recorded by comparing with the control embryos in a body length (after hatching, at and onwards 72 hpf) by optical comparison using an inverted microscope (CKX41; Olympus, Tokyo, Japan).
[f] Skin depigmentation of hyperpigmentation was assessed by optical comparison.
[g] Change in stellate morphology of the skin melanocytes was visually recorded

Table S4. List of antibodies used in this study.

Antibody	Target	Host	Company	Catalogue number	Assay
Anti-CD4 APC-eFluor™ 780	Human	Mouse IgG1, κ	Invitrogen	47-0049-41	FC
Anti-CD25 PE	Human	Mouse IgG1, κ	eBioscience	12-0259-80	FC
Anti-CD4 eF450	Mouse	Rat IgG2b, κ	eBioscience	48-0041-82	FC
Anti-CD4 FITC	Mouse	Rat IgG2b, κ	eBioscience	11-0041-85	FC
Anti-CD25 Alexa Fluor™ 488	Mouse	Rat IgG1, λ	eBioscience	53-0251-82	FC
Anti-CD25 PE	Mouse	Rat IgG1, κ	eBioscience	12-0251-82	FC
Anti-CTLA-4 FITC	Mouse	Armenian hamster IgG1	eBioscience	HMCD15201	FC
Anti-CD39 PE-Cy7	Mouse	Rat IgG2b, κ	eBioscience	25-0391-82	FC
Anti-PD-1 FITC	Mouse	Armenian hamster IgG1	eBioscience	11-9985-81	FC
Anti-CD73 PerCP-eFluor™ 710	Mouse	Rat IgG1	eBioscience	46-0731-80	FC
Anti-F4/80 Alexa Fluor™ 488	Mouse	Rat IgG2a	eBioscience	53-4801-82	FC
Anti-CD40 PE	Mouse	Rat IgG2a, κ	eBioscience	12-0401-81	FC
Anti-CD206 APC	Mouse	Rat IgG2a, κ	Biolegend	141708	FC
Anti-IL-10 FITC	Mouse	Rat IgG2b, κ	ThermoFisher Scientific	11-7101-82	FC
Anti-IL-17 FITC	Mouse	Rat IgG2a, κ	ThermoFisher Scientific	11-7177-81	FC
Anti-IFN-γ APC	Mouse	Rat IgG1, κ	ThermoFisher Scientific	17-7311-82	FC
Anti-FoxP3 PE	Mouse	Rat IgG2a, κ	ThermoFisher Scientific	12-5773-80	FC
Anti-FoxP3 APC	Mouse/ Human	Mouse IgG1, κ	Invitrogen	MA5-44084	FC
Anti-Ki-67 FITC	Mouse	Rat IgG2a, κ	ThermoFisher Scientific	11-5698-82	FC
Donkey Anti-Rabbit IgG H&L Alexa Fluor® 488	Mouse	Donkey IgG	Abcam	ab150073	FC/IF
Donkey-anti-mouse antibody Alexa Fluor 488	Mouse	Donkey IgG	Abcam	ab150105	IF
AHR	Mouse	Mouse	Invitrogen	MA1-513	IF

STAT3	Mouse	Rabbit	Cell Signaling Technology	4904s	WB
phosphoSTAT3	Mouse	Rabbit	Invitrogen	PA5-85801	WB
CYP1A1	Mouse	Rabbit	Invitrogen	PA5-101307	WB/FC/IF
β-Actin	Mouse	Rabbit	Abcam	ab8227	WB
Secondary antibody	Mouse	Goat	Invitrogen	62-6520	WB
Secondary antibody	Rabbit	Goat	Cell Signaling Technology	#7074	WB

FC – flow cytometry; IF – immunofluorescence; WB – western blot.

Table S5. List of primers used for qRT-PCR.

Gene	Species	Forward Sequence (5'-3')	Reverse Sequence (5′-3′)
Cyp1a1	Mouse	CAGACCTCAGCTGCCCTATC	TAACCTGCCACTGGTTCACA
β-actin	Mouse	GACCTGACAGACTACC	GGCATAGAGGTCTTTACGG
AHR	Human	CAAATCCTTCCAAGCGGCATA	CGCTGAGCCTAAGAACTGAAAG
AHRR	Human	GCGCCTCAGTGTCAGTTACC	CTCCTGCACGACTTGGAAGAA
CYP1A1	Human	ACATGCTGACCCTGGGAAAG	GGTGTGGAGCCAATTCGGAT
CYP1B1	Human	GGGACCGTCTGCCTTGTATG	GGTGGCATGAGGAATAGTGACA
GAPDH	Human	CATGAGAAGTATGACAACAGCCT	AGTCCTTCCACGATACCAAAGT
